# Clinical and Radiographic Outcomes of Vital Pulp Therapy Using Resin-Modified Versus Conventional Calcium Silicate-Based Materials: A Systematic Review and Meta-Analysis

**DOI:** 10.3390/jfb17010032

**Published:** 2026-01-07

**Authors:** Alberto Cabrera-Fernández, Laura Dominguez-Dominguez, Antonio Pérez-Pérez, João Miguel Marques Santos, Aránzazu Díaz-Cuenca, Daniel Torres-Lagares, Diana B. Sequeira, Juan J. Segura-Egea, Jenifer Martín-González

**Affiliations:** 1 Department of Stomatology, Endodontic Section, School of Dentistry, University of Sevilla, 41009 Sevilla, Spain; acabrera5@us.es (A.C.-F.); lauradguez96@gmail.com (L.D.-D.); danieltl@us.es (D.T.-L.); 2 Department of Medical Biochemistry and Molecular Biology and Immunology, School of Medicine, University of Seville, 41004 Seville, Spain; aperez14@us.es; 3 Institute of Endodontics, Faculty of Medicine, University of Coimbra, 3000-075 Coimbra, Portugal; jsantos@fmed.uc.pt (J.M.M.S.); disequeira@gmail.com (D.B.S.); 4 Materials Science Institute of Seville (ICMS), Joint CSIC-University of Seville Center, 41092 Sevilla, Spain; aranzazu@icmse.csic.es

**Keywords:** vital pulp therapy, resin-modified calcium silicate-based materials, TheraCal LC, TheraCal PT, meta-analysis, dentine bridge formation

## Abstract

Vital pulp therapy (VPT) is increasingly recognised as a biologically driven alternative to root canal treatment in teeth with deep caries and a vital pulp diagnosis. Resin-modified calcium silicate-based materials (RM-CSMs) were introduced to combine the bioactivity of traditional cements with improved handling and immediate light-curing, but their biological performance remains debated. Objectives: This systematic review and meta-analysis aimed to evaluate the clinical and radiographic outcomes of VPT performed with RM-CSMs compared with conventional non-resin-modified calcium silicate-based materials (NRM-CSMs) Methods: PRISMA Guidelines were followed to carry out this systematic review. Electronic databases (Medline, Embase, Scopus, and Web of Science) were searched up to October 2025 for randomised clinical trials evaluating indirect pulp capping, direct pulp capping, or pulpotomy. Nine trials met the inclusion criteria. Meta-analyses were performed for TheraCal LC, the only RM-CSM with sufficient clinical evidence. The risk of bias was assessed using the RoB 2 Tool. The certainty of evidence was assessed using GRADE. Results: Pooled results showed no significant differences in overall clinical–radiographic success between RM-CSMs and NRM-CSMs at 90 or 180 days. At 360 days, a trend favouring NRM-CSMs emerged, though not statistically significant. Dentine bridge formation at 360 days was significantly lower with TheraCal LC. Conclusions: Current RM-CSMs demonstrate comparable short-term success to conventional materials but still present biological limitations, particularly regarding long-term reparative outcomes. NRM-CSMs remain the preferred option when maximal bioactivity and predictable dentinogenesis are required

## 1. Introduction

Over recent decades, endodontics has shifted toward a more conservative paradigm focused on preserving the structural and biological integrity of the dentin–pulp complex whenever possible [1]. Within this context, vital pulp therapy (VPT) has gained prominence as a biologically driven approach aimed at controlling pulpal inflammation, maintaining pulp vitality in teeth diagnosed with pulpitis, and promoting tissue repair through the formation of a hard-tissue barrier [2]. Consequently, VPT represents a key strategy for preserving pulp function while supporting the long-term integrity of the tooth.

Traditionally, vital pulp therapy (VPT) was recommended primarily for mature permanent teeth diagnosed with reversible pulpitis. However, the principles of minimally invasive dentistry, together with growing scientific evidence, have expanded its indications to include cases of moderate and even severe pulpitis. As a result, VPT now encompasses a continuum of clinical procedures, including indirect pulp capping, direct pulp capping, partial pulpotomy, and full pulpotomy, each tailored to manage progressively advanced inflammatory conditions while preserving pulp vitality [3].

From a biological perspective, the success of VPT relies on the inherent defensive and reparative capacity of the dentin–pulp complex [4]. In response to mild stimuli, surviving odontoblasts upregulate matrix secretion and deposit reactionary dentin, whereas more severe injuries leading to odontoblast loss activate resident pulp stem cells, which can differentiate into odontoblast-like cells and produce reparative tertiary dentine [5]. Consequently, predictable healing depends on three key elements: (1) a favourable systemic and local environment, (2) effective control of bacterial contamination, and (3) the use of bioactive materials capable of supporting stem cell recruitment and differentiation [2].

In this regard, the development of calcium silicate-based materials (CSMs) has played a pivotal role in improving VPT outcomes [6]. Since the first introduction into dental research in 1993 [7], these materials have attracted substantial interest owing to their bioactivity, sealing performance, antimicrobial potential, and biocompatibility, ultimately establishing themselves as the reference standard for VPT [8]. Although ProRoot MTA, introduced in 1998, represented the first widely available formulation, its prolonged setting time and reliance on bismuth oxide, associated with discoloration and cytotoxic effects, posed significant limitations [9].

Subsequent advances in material science have led to improved formulations designed to overcome these drawbacks. Biodentine, introduced in 2011, exhibited enhanced mechanical and biological properties with encouraging clinical performance [10,11]. Nevertheless, handling difficulties, particularly its limited adhesion to dentin, remain a persistent clinical challenge [12].

To address these shortcomings, resin-modified calcium silicate-based materials (RM-CSMs) were developed. TheraCal LC [13]., introduced in 2011, is a light-curable hybrid material containing 30–50% calcium silicate. Despite acceptable physicochemical properties [14], multiple in vitro, in vivo, and clinical studies have questioned its biological performance, reporting inflammatory responses and reduced biocompatibility [15,16,17]. Consequently, a 2022 systematic review recommended its use only for indirect pulp capping and discouraged its application in direct pulp capping [18].

More recently, additional clinical trials with longer follow-ups have been published, and in 2017 a second-generation resin-modified material, TheraCal PT, became available [19], aiming to address the limitations of its predecessor.

Despite these developments, the existing evidence remains fragmented. The only previous systematic review addressing these materials focused largely on TheraCal LC and did not incorporate recently published clinical trials or newly introduced formulations such as TheraCal PT [18]. Moreover, that review did not consistently compare resin-modified materials with conventional bioceramics across the full range of vital pulp therapy (VPT) procedures, leaving an important gap in the current literature.

Given that concerns regarding the biocompatibility and pulpal response of resin-modified materials should ultimately manifest—positively or negatively—in clinical and radiographic outcomes, it is essential to synthesise the most recent evidence to determine whether these biological differences translate into meaningful clinical effects [20]. Clinical and radiographic outcomes represent the most relevant indicators of therapeutic success, as they reflect symptom resolution, pulpal healing, and long-term tissue stability, and directly inform clinical decision-making regarding the selection of the most appropriate material for maintaining pulpal vitality [21].

Despite the introduction of newer resin-modified materials such as TheraCal PT, the number of available randomised clinical trials remains limited. Consequently, although the present review aims to assess all RM-CSM formulations, the quantitative synthesis may be restricted to those materials for which sufficient clinical evidence exists

Given the emerging and, at times, conflicting evidence regarding the clinical performance of RM-CSMs, a comprehensive and methodologically rigorous synthesis is required to support informed clinical decision-making. Accordingly, the objective of this systematic review and meta-analysis is to evaluate the clinical and radiographic outcomes of vital pulp therapy performed using resin-modified calcium silicate–based materials compared with conventional non–resin-modified calcium silicate–based bioceramics (NRM-CSMs).

## 2. Materials and Methods

### 2.1. Review Question

This review was structured according to the Population–Intervention–Comparator–Outcome (PICO) framework.

Population (P): Permanent teeth with deep carious lesions and a vital pulp diagnosis, including cases of reversible pulpitis as well as symptomatic irreversible pulpitis treated with vital pulp therapy (VPT) procedures in accordance with contemporary diagnostic and therapeutic criteria.

Intervention (I): Resin-modified calcium silicate-based materials (RM-CSMs) used for VPT, such as TheraCal LC and TheraCal PT.

Comparator (C): Conventional resin-free calcium silicate-based materials (NRM-CSMs), including Biodentine, ProRoot MTA, NeoMTA, iRoot BP Plus, CEM, and similar materials.

Outcomes (O): Primary outcome: Combined clinical and radiographic success at 3–36 months, defined as absence of symptoms, maintenance of pulp vitality, and absence of radiographic pathology. Secondary outcomes included dentine bridge formation when reported.

Review question: In permanent teeth requiring vital pulp therapy due to deep caries and a vital pulp diagnosis (reversible or irreversible pulpitis), do resin-modified calcium silicate-based materials achieve clinical and radiographic success rates comparable to those obtained with conventional resin-free calcium silicate-based materials?

### 2.2. Search Strategy

Electronic databases (Medline, Embase, Scopus, and Web of Science) were systematically searched from their inception through October 2025 without language restrictions. Complete search strategies and database-specific modifications are detailed in Table 1. Grey literature was additionally examined, including screening reference lists of included studies, reviewing related systematic reviews, and contacting domain experts, to mitigate potential publication bias.

### 2.3. Inclusion and Exclusion Criteria

The inclusion and exclusion criteria used are described in Table 1.

### 2.4. Study Selection

The study selection process was conducted in accordance with PRISMA guidelines. All records retrieved from the database searches were imported into the Rayyan platform (Rayyan, Qatar Computing Research Institute, Qatar Foundation) for duplicate removal and screening. Four reviewers (A.C.-F., L.D-D., J.M-G., and J.J.S-E.) contributed to the selection process. A protocol was prospectively preregistered at the International Prospective Register of Systematic Reviews (PROSPERO) (CRD. 1249194).

Title and abstract screening was performed independently by two reviewers, who evaluated each record against the predefined eligibility criteria. Studies were considered potentially eligible when they involved permanent teeth with deep carious lesions and a vital pulp diagnosis, either reversible pulpitis or symptomatic irreversible pulpitis, managed using contemporary vital pulp therapy (VPT) procedures. Trials were included when they compared resin-modified calcium silicate-based materials (RM-CSMs) with at least one conventional resin-free comparator (NRM-CSMs) and reported combined clinical and radiographic outcomes with a minimum follow-up of 3 months.

Full texts were retrieved for all studies that met the initial screening criteria or provided insufficient information. Two reviewers independently assessed each full-text article for final eligibility. Exclusion reasons included: non-randomised or observational study design, use of non-qualifying materials, population limited to primary teeth, indication for root canal treatment rather than VPT, absence of an appropriate comparator, or insufficient reporting of clinical and radiographic outcomes within the 3–36-month range.

**Figure 1:**
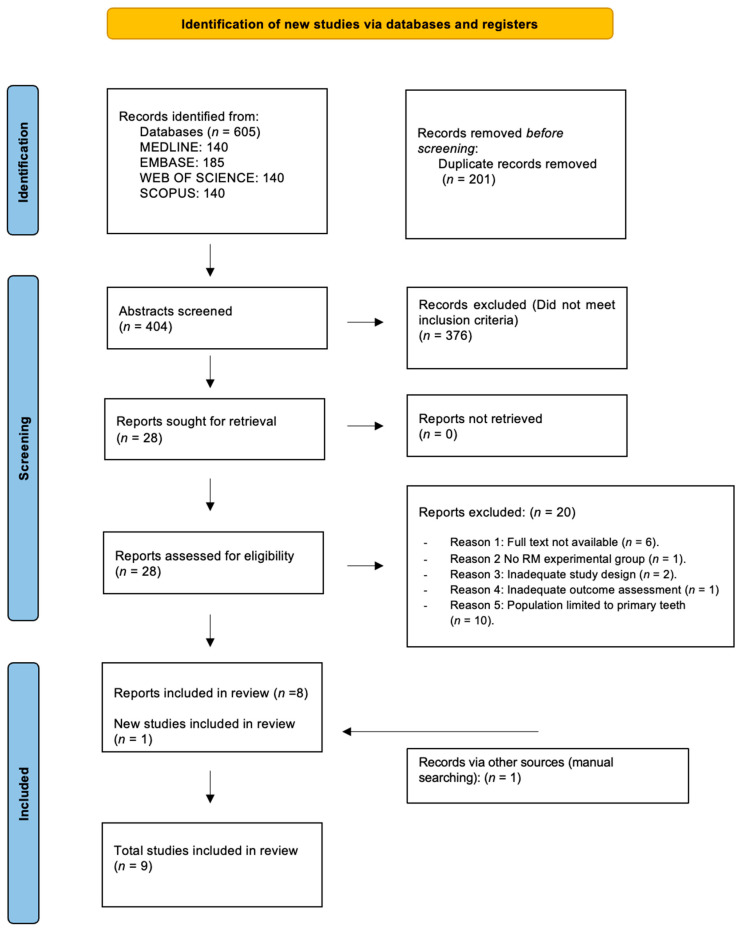
Search flowchart according to the PRISMA Statement.

Disagreements at any stage were resolved through discussion, with a third reviewer consulted when necessary. Mendeley Desktop (version 1.19.8; Elsevier Inc., New York, NY, USA) was used to manage references and organise the final set of included studies. The complete selection process is illustrated in the PRISMA flow diagram (Figure 1).

### 2.5. Data Extraction

Data extraction was carried out independently by two reviewers (A.C.F. and J.M.M.-S.) using a standardised and pilot-tested extraction form. For each included randomised clinical trial, the reviewers recorded: first author and publication year, sample size, participants’ clinical diagnosis, type of vital pulp therapy (VPT) procedure performed, follow-up duration, materials evaluated in the experimental (RM-CSM) and control (NRM-CSM) groups, definitions of clinical and radiographic success, and reported outcomes, including dentine bridge formation when available.

Any discrepancies between the two reviewers were resolved through discussion, and when necessary, by consulting a third reviewer (J.J.S.-E.). Full texts were revisited to clarify uncertain or missing information. When essential outcome data were not reported, corresponding authors were contacted. Studies for which outcome data could not be obtained were excluded from the quantitative synthesis for the affected outcomes. All extracted data were subsequently cross-checked for accuracy and consistency prior to analysis.

### 2.6. Quality Assessment and Risk of Bias of Individual Studies

The risk of bias of the randomised clinical trials included in this review was assessed using the revised Cochrane Risk of Bias tool for randomised trials (RoB 2) [22]. Two reviewers (L.D-D and J.M-G) independently evaluated each study according to the RoB 2 framework, which considers five domains related to internal validity: the randomization process, deviations from intended interventions, missing outcome data, measurement of the outcome, and selection of the reported result. The signalling questions within each domain were used to guide judgements, resulting in a domain-level rating of low risk of bias, some concerns, or high risk of bias. Any disagreements between the two reviewers were resolved through discussion or, when necessary, by consulting a third reviewer (A.C.-F.).

Following the RoB 2 decision rules, each study received an overall risk of bias judgement derived from the highest level of concern observed across its domains. Studies for which all domains were judged to be at low risk were classified as having an overall low risk of bias. Studies presenting some concerns in at least one domain, but without any domain rated as high risk, were categorised as having some concerns. Finally, any study judged to have a high risk of bias in at least one domain, or with multiple domains raising some concerns, was considered to be at high risk of bias.

The results of the risk of bias evaluation are illustrated using RoB 2 traffic-light and weighted-summary plots generated through the standard visualisation tool, providing a comprehensive overview of the methodological quality of the included trials.

### 2.7. Quality of Evidence

The certainty of the evidence for each outcome was assessed using the Grading of Recommendations Assessment, Development and Evaluation (GRADE) approach, implemented through the GRADEpro Guideline Development Tool (GRADEpro GDT; McMaster University, Hamilton, ON, Canada). Two reviewers (J.M.M.S. and J.J.S.-E.) independently evaluated the certainty of the evidence across the five core GRADE domains: risk of bias, inconsistency, indirectness, imprecision, and potential publication bias. Any disagreements were resolved through discussion, and when necessary, by consulting a third reviewer (D.B.L-S).

As per GRADE methodology, the certainty of evidence for each outcome was initially rated as high because all included studies were randomised clinical trials. The certainty rating was subsequently downgraded when concerns were identified in one or more domains. Downgrading decisions considered the following: (1) study-level methodological limitations identified in the RoB 2 assessment; (2) inconsistency of effect estimates or substantial statistical heterogeneity; (3) indirectness related to population, interventions, or outcomes that did not fully align with the review question; (4) imprecision stemming from wide confidence intervals or small sample sizes; and (5) possible publication bias based on asymmetry or scarcity of evidence.

Final certainty ratings were classified as high, moderate, low, or very low according to GRADE criteria. Summary of Findings (SoF) tables were generated in GRADEpro to provide an explicit and transparent overview of the certainty of evidence for each primary and secondary outcome included in the quantitative synthesis.

### 2.8. Quantitative Analysis (Meta-Analysis)

A quantitative synthesis was performed for outcomes that were reported in a sufficiently comparable manner across studies. For each randomised clinical trial, clinical and radiographic success rates were extracted. Studies were grouped by both follow-up duration and VPT modality, and meta-analyses were conducted only when at least two studies contributed data to the same comparison. Peskersoy et al. (2021) reported clinical and radiographic success independently, so they were not included in our meta-analysis [23].

All analyses were carried out using Review Manager (RevMan) version 5.4. Owing to the expected clinical and methodological heterogeneity among trials, a random-effects model was applied in all cases. For dichotomous outcomes, the risk ratio (RR) and its 95% confidence interval (CI) were used as the summary effect measure. Statistical heterogeneity was evaluated using the I^2^ statistic, and statistical significance was set at *p* < 0.05. Pooled estimates were calculated using the Mantel–Haenszel method in accordance with Cochrane guidelines.

Although the review aimed to include all available resin-modified calcium silicate-based formulations, the number of eligible randomised clinical trials was highly uneven across materials. In particular, TheraCal PT, despite being a newer formulation, had an insufficient number of clinical studies meeting the inclusion criteria. Therefore, quantitative synthesis was restricted to the material with adequate evidence, TheraCal LC.

All primary meta-analyses compared TheraCal LC (experimental) against non-resin-modified calcium silicate-based materials (control). When possible, subgroup meta-analyses were carried out to evaluate specific material-level contrasts. These subgroup analyses were limited to the comparison of TheraCal LC versus Biodentine, which was the only control material consistently represented across multiple studies.

In addition to the primary analyses of clinical and radiographic success, supplementary meta-analyses were performed to assess dentine bridge formation, pooling all available control materials. These analyses were conducted for the 180-day and 360-day follow-up intervals.

Finally, to increase the number of studies available for quantitative synthesis, an additional meta-analysis was performed combining direct pulp capping (DPC) and pulpotomy procedures. When quantitative pooling was not feasible due to insufficient data, results were reported narratively.

## 3. Results

### 3.1. Bibliographic Search (Study Selection)

Figure 1 shows the PRISMA 2020 flow diagram summarising the search strategy and study selection process. The initial search across all databases yielded 605 records. After removing duplicates, 404 unique studies remained. Following title and abstract screening, 376 of these were excluded for not meeting the inclusion criteria or study objectives.

A full-text assessment was conducted for 28 articles, of which 20 were excluded for the following reasons:

- Full text not available (*n* = 6).
- No resin-modified experimental group (*n* = 1).
- Inadequate study design (retrospective observational) (*n* = 2).
- Inadequate assessment of clinical or radiographic success (*n* = 1).
- Population restricted to primary teeth (*n* = 10).

Additionally, one further study was identified through hand-searching.

Finally, A total of 9 randomised controlled trials met the inclusion criteria and were included in the qualitative synthesis ([20,23,24,25,26,27,28,29,30]).

### 3.2. Qualitative Synthesis (Descriptive Summary)

**Figure 2:**
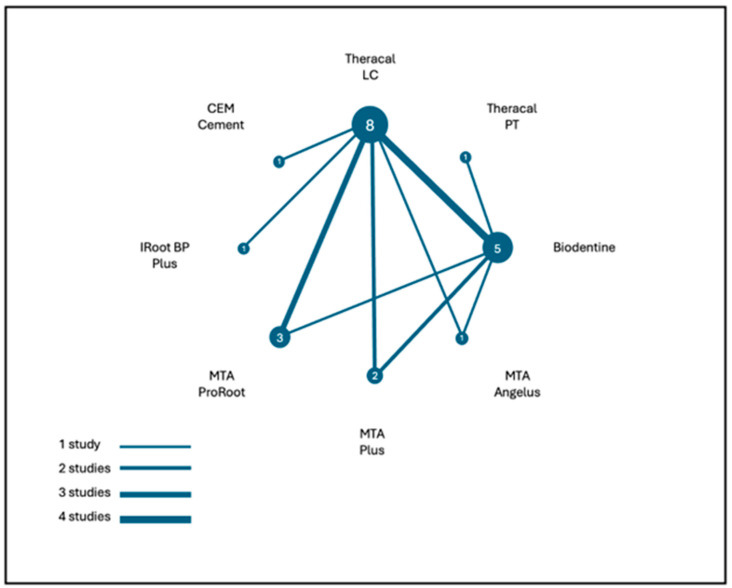
The network diagram of pairwise comparisons among the materials evaluated across the included trials. The node size reflects the number of studies using each material, and the edge thickness indicates the number of direct comparisons.

Figure 2 displays a network diagram illustrating all direct pairwise comparisons between resin-modified and non–resin-modified CSMs. Each node represents a biomaterial evaluated in the included trials, while the connecting edges (labelled by the number of studies) indicate how frequently each comparison was investigated.

Table 2 summarises the main characteristics of the included studies. Two RM-CSMs were identified: TheraCal LC, evaluated across multiple vital pulp therapy (VPT) procedures (IPC, DPC, and pulpotomy), and TheraCal PT, which was assessed in a single study exclusively for pulpotomy [29]. The follow-up periods among TheraCal LC trials ranged from 21 days to 5 years, whereas the study evaluating TheraCal PT reported follow-ups between 90 and 360 days. Across all trials, sample sizes varied between 22 and 525 teeth.

Control groups (NRM-CSM) included CEM Cement, Biodentine, ProRoot MTA, and iRoot BP Plus, with Biodentine being the most frequently employed comparator. Regarding clinical diagnosis, all studies included teeth with reversible pulpitis, except Zhang et al., 2024 [20], which also enrolled cases of irreversible pulpitis in the context of full pulpotomy. All trials assessed overall treatment success, defined as a combination of clinical and radiographic criteria, with the exception of Peskersoy et al. (2021) [23], which reported clinical success and radiographic success as separate outcomes.

Although individual definitions varied slightly across studies, global success criteria were broadly comparable and typically encompassed the absence of spontaneous pain, absence of tenderness to percussion, absence of swelling, sinus tract or fistula, positive response to pulp vitality testing, and absence of radiographic signs of periapical or furcal pathology. In addition to global success rates, five studies [23,26,28,29] also reported outcomes related to dentine bridge formation.

### 3.3. Meta-Analysis

**Figure 3:**
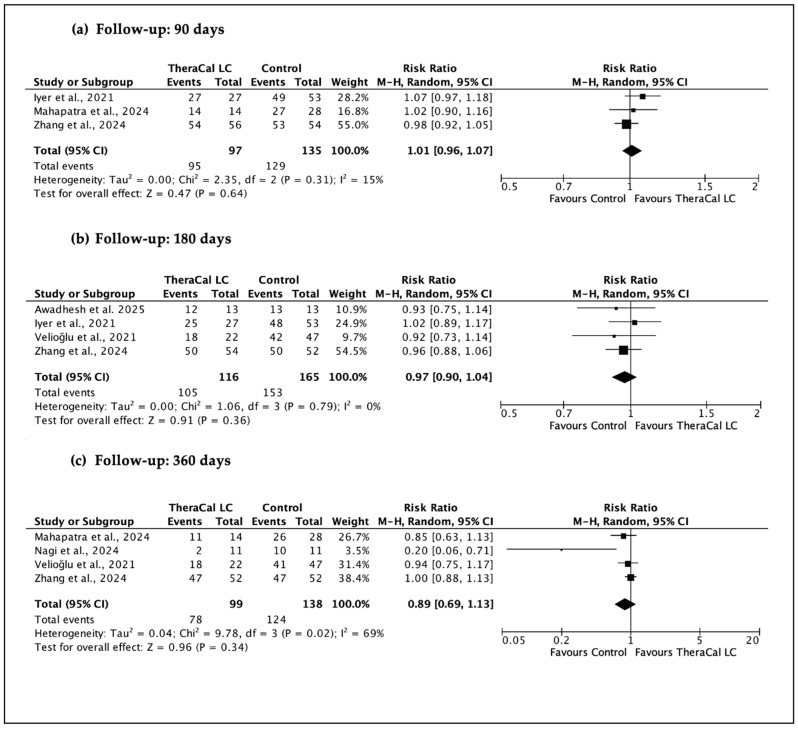
Meta-analyses of clinical and radiographic success of vital pulp therapy procedures (IPC, DPC or Pulpotomy) using resin-modified calcium silicate-based material (Theracal LC) versus non-resin-modified controls at 90, 180, and 360 days. (**a**) [20,26,28], (**b**) [20,25,26,30], (**c**) [20,25,27,28].

**Figure 4:**
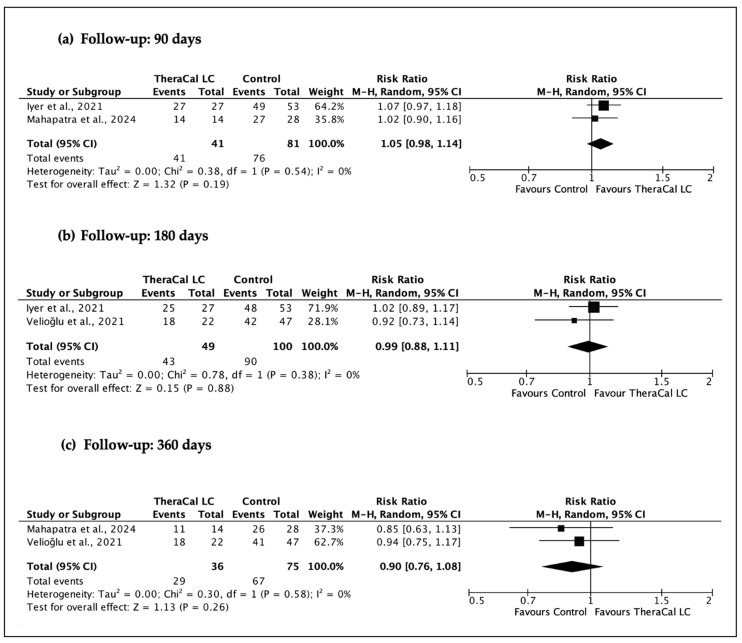
Meta-analyses of clinical and radiographic success of direct pulp capping using resin-modified CSM (TheraCal LC) versus non-resin-modified controls at 90, 180, and 360 days. (**a**) [26,28], (**b**) [25,26], (**c**) [25,28].

**Figure 5:**
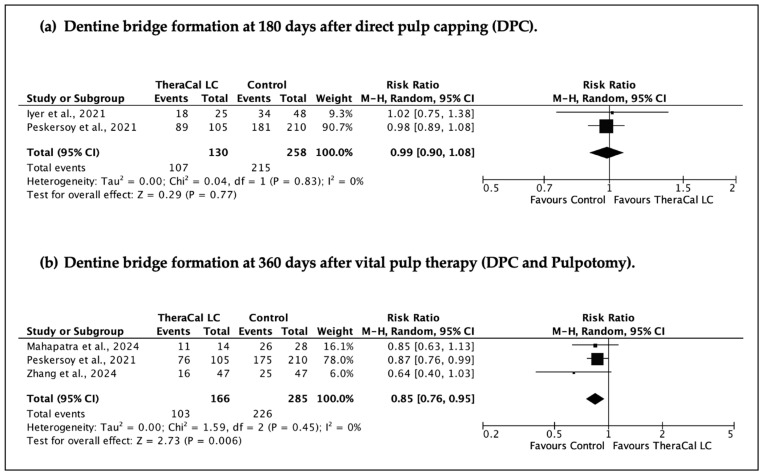
Meta-analysis of dentine bridge formation following the use of resin-modified versus conventional resin-free calcium silicate-based materials. (**a**) [23,26], (**b**) [20,23,28].

**Figure 6:**
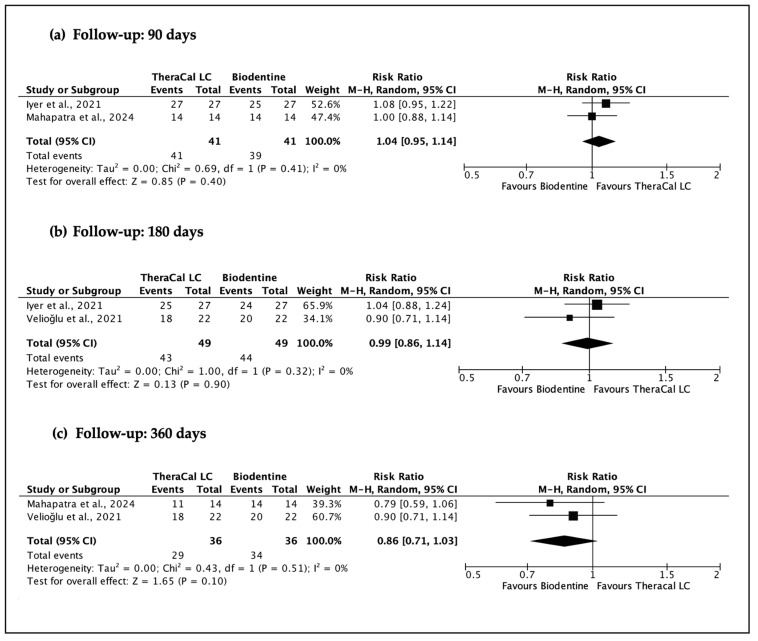
Subgroup meta-analyses of clinical and radiographic success of direct pulp capping using resin-CSM (TheraCal LC) versus Biodentine at 90, 180, and 360 days. (**a**) [26,28], (**b**) [25,26], (**c**) [25,28].

A total of four sets of meta-analyses were performed (Figure 3, Figure 4, Figure 5 and Figure 6), evaluating the clinical and radiographic success of resin-modified calcium silicate-based materials (RM-CSMs) in comparison with non-resin-modified materials (NRM-CSMs) across different VPT procedures and follow-up periods.

Across all studies combined (Figure 3), the pooled analysis at 90 days showed no significant difference in overall clinical and radiographic success between RM-CSMs and NRM-CSMs (RR ≈ 1.01, 95% CI 0.94–1.07), with minimal heterogeneity (I^2^ = 15%). Similar findings were observed at 180 days (RR ≈ 0.97, 95% CI 0.90–1.04, I^2^ = 0%) and 360 days (RR ≈ 0.89, 95% CI 0.69–1.13, I^2^ = 69%). These results indicate a consistent pattern: resin-modified materials do not exhibit inferior or superior success compared with traditional bioceramics, and the stability of I^2^ = 0–15% across 90 and 180 days reinforces the robustness and agreement of the included trials.

When restricting the analysis specifically to direct pulp capping (DPC) procedures (Figure 4), similar conclusions were obtained. At 90 days, the pooled effect was RR 1.05 (95% CI 0.98–1.14); at 180 days, RR 0.99 (95% CI 0.88–1.11); and at 360 days, RR 0.90 (95% CI 0.76–1.08). Again, all analyses showed I^2^ = 0%, confirming little variability across trials and reinforcing the absence of detectable clinical differences between resin-modified and non-resin-modified materials within the context of DPC.

Analysis of dentine bridge formation (Figure 5) yielded a slightly different trend. At 180 days, no significant differences were observed (RR 0.99, 95% CI 0.90–1.08, I^2^ = 0%), indicating similar early dentinogenic potential between RM-CSMs and NRM-CSMs. However, at 360 days, the pooled result showed a significant advantage for conventional materials, with RM-CSMs demonstrating lower rates of complete dentine bridge formation (RR 0.85, 95% CI 0.76–0.95, I^2^ = 0%).

A predefined subgroup meta-analysis comparing TheraCal LC specifically against Biodentine (Figure 6) corroborated the general findings. At 90 days (RR 1.04, 95% CI 0.95–1.14) and 180 days (RR 0.99, 95% CI 0.86–1.14), no material showed clear superiority. At 360 days, the effect favoured Biodentine (RR 0.86, 95% CI 0.71–1.03), although the confidence interval crossed unity and therefore remained statistically non-significant. The consistent I^2^ = 0% again suggests strong agreement between studies and reliability of estimates.

### 3.4. Risk of Bias of Included Studies

**Figure 7:**
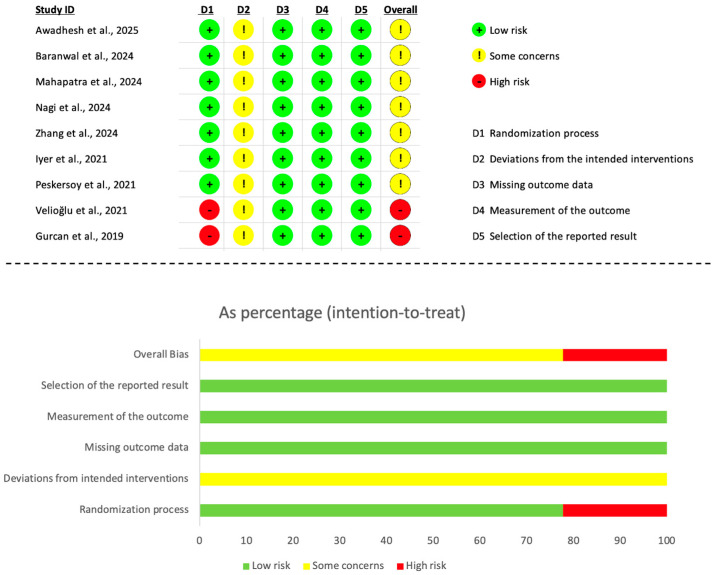
Risk of bias of the included trials according to the RoB 2 tool [20,23,24,25,26,27,28,29,30].

The risk of bias of the included randomised clinical trials was assessed using the RoB 2.0 tool (Figure 7). Overall, most studies were judged as presenting some concerns, while two trials [24,25] were rated at high risk of bias. The randomisation process (Domain 1) was adequately described and appropriately implemented in the majority of studies, resulting in a low-risk judgement. In contrast, Velioğlu et al. and Gurcan et al. provided insufficient or inconsistent information regarding sequence generation and allocation concealment, leading to a high-risk rating for this domain 1.

All included studies were judged as having some concerns in Domain 2, which assesses deviations from the intended interventions. This consistent finding reflects an inherent limitation in VPT trials: blinding of operators is not feasible due to the easily recognisable handling and visual characteristics of calcium silicate-based materials.

Regarding missing outcome data (Domain 3), attrition was minimal, balanced between groups, and generally well explained, resulting in a low-risk judgement for all studies. Similarly, all trials were assessed as low risk in Domain 4, as clinical and radiographic outcomes were measured using objective and standardised criteria.

Finally, all studies were judged as low risk in Domain 5 (selection of the reported result), with no evidence of selective reporting or inconsistencies between prespecified outcomes and reported findings. The distribution of risk-of-bias judgements across domains is presented in Figure 7.

Taken together, the overall risk of bias was judged as some concerns for the majority of included trials, mainly driven by the unavoidable inability to blind operators in this type of intervention.

### 3.5. GRADE Assessment of Quality of Evidence

The certainty of the evidence was evaluated according to the GRADE (Grading of Recommendations Assessment, Development and Evaluation) approach, which considers five domains: risk of bias, inconsistency, indirectness, imprecision, and publication bias. As illustrated in Table 3, the overall certainty of the evidence supporting the comparison between TheraCal LC (resin-modified calcium silicate-based) and the non-resin-modified calcium silicate-based controls was rated as low.

The initial certainty of the evidence started as high, as all included studies were randomised controlled trials. However, the certainty was downgraded due to methodological limitations and imprecision in the pooled estimates.

First, the risk of bias was rated as serious, since several trials presented some concerns across multiple RoB2 domains, particularly in the randomization process. Two studies showed a high risk of bias, further reducing confidence in the internal validity of the evidence.

By contrast, inconsistency was not considered a serious limitation. The direction and magnitude of effect estimates were largely consistent across studies and follow-up periods (90, 180, and 360 days), with all pooled-risk ratios centred around 1.0 and showing no meaningful clinical heterogeneity between trials evaluating resin-modified versus non–resin-modified calcium silicate-based materials.

However, imprecision was judged to be serious, as reflected by wide confidence intervals that crossed the line of no effect in all primary analyses. Although the point estimates showed minimal differences between materials, the confidence intervals allowed for both potential benefit and potential harm. Additionally, most meta-analyses included a small number of participants and did not meet the optimal information size, further contributing to imprecision.

Indirectness and publication bias were not considered serious concerns, given that all trials directly compared resin-modified and conventional calcium silicate-based materials in vital pulp therapy procedures using clearly defined clinical and radiographic outcomes.

Taken together, although the evidence suggests no clinically important difference in treatment success between resin-modified and non-resin-modified calcium silicate-based materials, the overall certainty of evidence was rated as low. This indicates that the true effect may differ meaningfully from the observed estimate, and further high-quality, adequately powered randomised controlled trials are needed.

## 4. Discussion

Our findings indicate that resin-modified calcium silicate-based materials (RM-CSMs), particularly TheraCal LC, which is the only material supported by sufficiently robust evidence, exhibit clinical and radiographic success rates in VPT that are comparable to conventional calcium silicate-based materials during the initial months of follow-up.

The analysed data show a similar trend between materials, with no statistically significant differences in overall success at 90, 180, or 360 days. However, as the follow-up period progresses, a gradual shift in the risk ratio can be observed in favour of non-resin-based formulations, although this trend does not reach statistical significance.

In contrast, the assessment of dentine bridge formation, one of the key biological indicators of reparative success [31,32,33], reveals significant differences (*p* = 0.006) at 360 days, with conventional calcium silicate-based materials demonstrating clear superiority.

In this meta-analysis, the primary outcome was overall success, defined as the combined achievement of clinical and radiographic success. Clinically, success is characterised by the absence of spontaneous pain, tenderness to percussion, swelling, sinus tract, or fistula, together with a normal response to pulp vitality testing. Radiographic success is defined as the absence of periapical or furcal radiolucency, periodontal ligament widening, or any signs of progressive pathology.

The use of this combined clinical–radiographic success measure is consistent with the methodology adopted in much of the vital pulp therapy (VPT) literature [3,34]. Likewise, current clinical guidelines, including those of the American Academy of Pediatric Dentistry (AAPD), describe treatment success in terms of both clinical and radiographic criteria [35]. This alignment across sources supports the validity of using an overall, combined success measure as the primary outcome in the present meta-analysis.

For consistency with this established framework, only studies reporting integrated clinical–radiographic outcomes were included in the quantitative synthesis. Trials presenting these outcomes separately were excluded from the meta-analysis to maintain methodological homogeneity.

This is the case of Peskersoy et al. (2021) [23], who reported clinical and radiographic success independently at 30, 180, 360, and 1080 days. Although these data could not be incorporated into the meta-analysis, their findings are consistent with the trends observed in our quantitative synthesis. Specifically, they show a progressive decline in the success rate of TheraCal LC, with an increasing difference compared with Biodentine and MTA Plus as the follow-up time lengthens.

These findings are consistent with the extensive body of literature supporting the clinical and biological performance of NRM-CSMs in vital pulp therapy [36,37]. These materials, composed primarily of Dicalcium Silicate (C_2_SiO_4_) and Tricalcium Silicate (Ca_3_SiO_5_) phases, have consistently demonstrated excellent sealing ability, antibacterial activity, and marked bioactivity [8].

Given the pivotal role that bioactivity plays in the clinical and biological performance of calcium silicate-based materials [38], it becomes essential to understand how the incorporation of resin affects this and other key properties. TheraCal LC is one of the most extensively studied resin-modified calcium silicate-based materials, and its physicochemical properties have been consistently characterised in the scientific literature. The material exhibits favourable performance in terms of compressive strength, solubility, porosity, and Ca^2+^ release, an essential factor underpinning bioactivity [39,40]. However, cytotoxicity appears to be its principal limitation and likely contributes to its comparatively reduced clinical performance over the medium and long term. In ex vivo entire tooth culture models, TheraCal LC has shown delayed and less-abundant formation of early mineralisation foci when compared with non-resin-modified materials such as ProRoot MTA or Biodentine [41,42], a finding that aligns with our results demonstrating a significantly lower rate of dentine bridge formation at 360 days. Likewise, several in vitro studies using dental pulp stem cells have reported significantly reduced cell viability in the presence of TheraCal LC compared with conventional CSM [43,44,45].

A key factor underlying this behaviour is the sustained release of unpolymerized resin monomers, which are known to induce cellular apoptosis. These hydrophobic components, primarily polyethyleneglycol dimethacrylate (PEGDMA) and bisphenol A-glycidyl methacrylate (Bis-GMA), can leach from the material due to incomplete polymerization and diffuse into the surrounding tissues. Once released, they increase oxidative stress, disrupt mitochondrial function, and compromise cell-membrane integrity, ultimately reducing cell viability and interfering with odontoblastic differentiation [46,47]. This mechanistic pathway has been repeatedly identified as a major biological limitation of Theracal LC.

To overcome the adverse effects associated with the release of unpolymerized resin monomers from light-cured formulations, TheraCal PT was developed as a dual-cure (chemical- and light-activated) CSM. According to the manufacturer, it is specifically indicated for indirect pulp capping (IPC), direct pulp capping (DPC), and pulpotomy procedures. However, this encouraging description may contrast with the available scientific evidence.

Recent in vivo research by Gök et al. (2025) [48] reported that TheraCal PT exhibited inferior performance in terms of inflammatory-cell infiltration and hard-tissue formation compared with Biodentine, particularly in teeth presenting pre-existing pulpal inflammation. At the cellular level, Novotná et al. (2024) demonstrated that eluates of TheraCal PT at higher concentrations induced measurable cytotoxicity. However, its cytotoxic effect remained consistently lower than that of TheraCal LC under the same conditions [49]. Comparable findings were reported by Küden et al. (2022), who observed cytotoxicity values for TheraCal PT similar to those of TheraCal LC and higher than those associated with MTA [50].

Nonetheless, other studies have described a more favourable biological profile for TheraCal PT relative to its predecessor. Rodríguez-Lozano et al. (2021) reported acceptable biocompatibility for TheraCal PT [51], and Park et al. (2024) found that it induced significantly greater hard-tissue formation, elicited a lower inflammatory response (reduced CD68 expression), and promoted higher DSPP expression, suggesting an enhanced capacity to stimulate dentinogenesis [52]. Similar conclusions were reached by Quiñónez et al. (2023) and Sanz et al. (2021), who also described improved biocompatibility of TheraCal PT compared with TheraCal LC [53,54].

In terms of bioactivity, Elbanna et al. (2022) reported that TheraCal PT exhibited lower Ca^2+^ release, reduced alkalinization potential, and inferior apatite-forming ability compared with Biodentine and even TheraCal LC, which may help explain its more modest biological behaviour in some studies [55].

The improved biological behaviour reported for TheraCal PT compared with TheraCal LC may be explained by its lower release of unpolymerised resin monomers, particularly Bis-GMA and PEGDMA, resulting from the higher degree of polymerisation achieved through its dual-cure setting mechanism [48]. In addition, TheraCal PT produces significantly fewer reactive oxygen species (ROS), which may help explain its improved cellular response compared to the previous material, Theracal LC [54]. Nevertheless, although its biological performance appears superior to that of TheraCal LC, it still does not match the outcomes consistently reported for conventional non-resin-modified calcium silicate-based materials [50].

From a clinical perspective, the present review identified only one randomised controlled trial in permanent teeth comparing TheraCal PT with a non-resin-modified calcium silicate-based material (Biodentine). The study by Baranwal et al. could not be included in the quantitative synthesis, as it was the single clinical trial evaluating this material; however, its findings must be considered within this descriptive synthesis [29]. TheraCal PT showed lower overall success rates and poorer dentine bridge formation than Biodentine at all follow-up periods (90, 180, and 360 days) when used in pulpotomy procedures. These results are consistent with the available in vitro evidence, which likewise indicates a more limited biological performance of TheraCal PT relative to conventional calcium silicate-based materials. Moreover, these data are similar to the quantitative synthesis conducted regarding the results of Theracal LC.

Other resin-modified calcium silicate-based materials have been commercialised, such as BioCal-CAP (Harvard) and Oxford ActiveCal CP. The present review did not identify any clinical trials evaluating these materials, and therefore, no data concerning their performance could be included in our synthesis. However, a small number of in vitro and animal studies provide preliminary insight into their biological behaviour.

In an in vivo zebrafish model [56], it was reported that both BioCal-CAP and Oxford ActiveCal CP exhibited lower toxicity and higher biocompatibility, even at elevated concentrations, when compared with the non-RM controls MTA Angelus and Biodentine. Similarly, an in vitro study by Tez et al. (2024) demonstrated favourable outcomes for BioCal-CAP, including low cytotoxicity (reduced ROS expression), acceptable biocompatibility, and promising angiogenic potential [57]. These findings may be partly explained by the absence of unreacted HEMA monomers in BioCal-CAP, a feature that distinguishes it from TheraCal LC [58].

However, despite these promising preliminary observations, the current evidence supporting alternative resin-modified calcium silicate-based materials, such as BioCal-CAP and Oxford ActiveCal CP remains very limited. Additional in vitro studies and, critically, human randomised clinical trials are required.

It is also important to highlight promising experimental developments, such as the work by Pedano et al. (2021), who designed an experimental resin-modified calcium silicate-based material (Exp_HAA) in which the conventional monomers Bis-GMA, HEMA and PEGDMA were replaced by alternative components, N-(2-hydroxyethyl) acrylamide (HEAA) and urethane dimethacrylate (UDMA) [59]. This formulation achieved superior outcomes in terms of bioactivity, dentine adhesion, and biocompatibility with human dental pulp stem cells. Moreover, when tested in an ex vivo natural tooth culture model, Exp_HAA demonstrated a greater ability to promote early dentinogenesis compared with TheraCal LC. Similar findings regarding improved cell viability were reported by Park et al. (2021), who developed a Bis-GMA-free RM-CSM exhibiting a more favourable cytocompatibility [60]. These outcomes suggest that optimisation of the resin matrix may substantially improve the biological performance of future RM-CSMs [59]

However, resin-modified calcium silicate-based materials also present advantages over conventional non-resin-modified formulations, particularly in relation to their mechanical properties. These materials have demonstrated favourable performance in terms of compressive strength, dimensional stability, and microhardness, attributes already reported for TheraCal LC [61] and further improved in TheraCal PT. In addition, RM-CSMs exhibit enhanced dentine adhesion [62] and better compatibility with resin composites compared with traditional non-resin materials [63].

Nevertheless, the most significant clinical contribution of RM-CSMs lies in their excellent handling characteristics. Their flowable consistency allows precise placement, particularly in deep cavities and pulp exposures, and immediate light-curing enables rapid stabilisation of the material, facilitating efficient, single-visit restorative procedures [61,63].

These practical advantages translate into clear clinical implications. RM-CSMs constitute a valid alternative in situations where reduced clinical time and simplified handling are essential. Such conditions are particularly relevant in paediatric dentistry, where patient cooperation is limited and treatment efficiency is critical, as well as in scenarios involving deep cavities or pulp exposures that require immediate and stable placement of the material [64]. However, considering our outcomes, when the clinical objective prioritises maximal bioactivity or a more robust long-term reparative response, conventional non-RM-CSMs remain the gold standard.

The low heterogeneity observed across most analyses supports the methodological consistency of the included studies. The differences detected in a small number of outcomes may be explained by variation in the types of vital pulp therapies evaluated, diagnostic discrepancies between reversible and irreversible pulpitis, operator-dependent technical differences, heterogeneity in the comparative materials used, and the coexistence of different resin-modified formulations. Subgroup analyses, particularly those limited to comparisons with Biodentine, showed a further reduction in heterogeneity, reinforcing the stability and reliability of the main findings.

This meta-analysis has several limitations that are inherent to the available evidence. First, the conclusions of the quantitative synthesis cannot be generalised to all resin-modified calcium silicate-based hydraulic bioceramics. Although several newer materials, such as TheraCal PT, BioCal-CAP, and Oxford ActiveCal CP, have been introduced, the available evidence for these formulations is insufficient to permit a meta-analysis. Consequently, the quantitative findings of the present review apply exclusively to TheraCal LC, which was the only resin-modified material supported by an adequate number of comparable clinical studies to allow a robust statistical evaluation.

For the studies included in the quantitative analysis, certain methodological constraints must be acknowledged. Some trials presented moderate or high risk of bias, relatively small sample sizes, and short follow-up periods, which limit the ability to fully assess long-term clinical and biological outcomes. Although diagnostic criteria for reversible and irreversible pulpitis, as well as definitions of success in vital pulp therapy, have become increasingly standardised, some residual variability persists between studies. Differences in operative techniques and restorative protocols may also contribute to the observed heterogeneity.

This meta-analysis focused exclusively on permanent teeth. While this approach avoids mixing populations with inherently different biological and anatomical characteristics, it restricts the applicability of the findings to permanent dentition. Primary teeth exhibit greater dentinal permeability, larger pulp chambers, a more accelerated inflammatory response, and distinct reparative dynamics. Therefore, the behaviour of resin-modified and non–resin-modified calcium silicate-based hydraulic bioceramics in primary dentition cannot be directly inferred from these results. Dedicated clinical research in primary teeth is required to establish their performance in paediatric dentistry [65,66].

Given these limitations, future research should prioritise well-designed randomised clinical trials directly comparing the main resin-modified calcium silicate-based hydraulic bioceramic materials currently available—TheraCal LC, TheraCal PT, BioCal-CAP, and Oxford ActiveCal CP—to clarify their relative clinical and biological performance. Long-term follow-ups of at least three to five years are necessary to assess true reparative stability over time. Further studies should incorporate standardised diagnostic criteria aligned with contemporary pulp disease classifications and include complementary biological outcomes, such as quantitative biomarker analysis or pulp vitality parameters. In addition, clinical testing in humans of emerging experimental resin-modified calcium silicate-based bioceramics, such as Bis-GMA–free formulations that have shown promising in vitro and ex vivo results, is essential to determine their translational potential.

## 5. Conclusions

Resin-modified calcium silicate-based materials represent a promising class of materials whose adoption has been driven primarily by their favourable handling properties, accelerated setting kinetics, and applicability in clinical situations characterised by operative complexity. These operational benefits justify their expanding role in vital pulp therapy and their consideration as alternatives in selected clinical indications.

Nonetheless, currently available formulations still exhibit significant biological constraints. The collective evidence indicates that resin-modified calcium silicate-based materials do not yet reproduce the biocompatibility, calcium-ion release, or bioactivity consistently achieved by conventional non–resin-modified calcium silicate-based materials, which continue to serve as the benchmark for optimal reparative performance.

Within this meta-analysis, TheraCal LC—the only resin-modified material supported by adequate clinical evidence—demonstrated short-term success rates comparable to those of traditional materials but failed to show superiority and exhibited limited capacity for dentine bridge formation. Taken together, the findings underscore that, while resin-modified calcium silicate-based materials offer important practical advantages, conventional non-resin-modified materials remain the most appropriate choice when the therapeutic objective is to maximise bioactivity, ensure predictable tissue repair, and promote durable pulpal healing.

## Figures and Tables

**Table 1:** Search strategy and eligibility criteria for the systematic search.

Component	Description
Databases searched	Medline, Scopus, Embase, Web of Science
Search period	October 2025
Search terms	((“calcium silicate” [tiab] OR “calcium silicate-based” [tiab] OR “hydraulic calcium silicate” [tiab] OR “mineral trioxide aggregate” [tiab] OR MTA [tiab]) AND (“resin-modified” [tiab] OR “resin-containing” [tiab] OR “light-cured” [tiab] OR “dual-cure” [tiab] OR photocur* [tiab] OR “TheraCal LC” [All Fields] OR “TheraCal PT” [All Fields] OR Theracal [All Fields] OR “Harvard BioCal” [All Fields] OR “BioCal-CAP” [All Fields] OR “Oxford ActiveCal” [All Fields])) NOT (“glass ionomer” [tiab] OR “calcium phosphate cement” [tiab] OR “phosphate cement” [tiab] OR “adhesive system” [tiab] OR “bond strength” [tiab] OR review [pt])
Language restrictions	No language restrictions
Inclusion criteria	- Only randomised clinical trials, using either a parallel or split-mouth design, were eligible for inclusion. - The study population had to consist of permanent teeth with deep carious lesions indicated for vital pulp therapy, including indirect pulp capping, direct pulp capping, or pulpotomy. - The intervention had to involve a resin-modified calcium silicate-based material, such as TheraCal LC, TheraCal PT, Activa BioActive-Liner, or BioCal-CAP. - At least one comparison group treated with a conventional, resin-free calcium silicate-based material (MTA, Biodentine, or NeoMTA) was required. - Eligible studies needed to provide clinical and radiographic outcome data with a follow-up duration between 3 and 36 months, at least. - Full-text availability was mandatory.
Exclusion criteria	- Non-relevant diagnosis or indication: Trials in which the diagnosis was pulp necrosis or severe pulpitis but the indicated treatment was conventional root canal therapy rather than vital pulp therapy. - Inadequate comparator or intervention: Trials that did not include a control group treated with a conventional resin-free calcium silicate-based material, or that did not include an experimental group treated with a resin-modified calcium silicate-based material. - Non-eligible study design: Studies that were non-randomised, quasi-randomised, observational, in vitro, or animal experiments. - Insufficient outcome data: Studies that did not report clinical and radiographic follow-up results within the 3- to 36-month range, at least.
Screening process	Titles and abstracts screened independently by two reviewers; full texts assessed for eligibility
Data extraction	Standardised extraction of sample size, VPT procedure, success criteria, follow-up time, materials tested, success outcomes, and dentine bridge formation.

**Table 2:** Data extraction of clinical success and dentine bridge formation.

First Author, Year Published	Sample, Age (Years)	Clinical Diagnosis	VPT Procedure	Follow up (days)	Material Tested	Success Outcomes (Clinical and Radiographic)								Dentine Bridge Formation			
Awadhesh et al., 2025 [30]	52 teeth, 17–40	Reversible pulpitis	IPC	21, 90, 180		**180d**								---			
					CEM Cement	13/13											
					TheraCal LC	12/13											
Baranwal et al., 2024 [29]	60 teeth, 18–40	Irreversible pulpitis	Pulpotomy	7, 90, 180, 360		**90d**		**180d**				**360d**		**90d**	**180d**		**360d**
					Biodentine	23/25		22/25				21/25		1/25	3/25		5/25
					TheraCal PT	18/22		17/22				17/22		0/22	2/22		4/22
Mahapatra et al., 2024 [28]	42 teeth, 17–40	Reversible pulpitis	DPC	21, 90, 360		**21d**		**90d**				**360d**		**21d**	**90d**		**360d**
					Biodentine	14/14		14/14				14/14		0/14	13/14		12/14
					MTA ProRoot	14/14		13/14				12/14		0/14	11/14		14/14
					TheraCal LC	14/14		14/14				11/14		0/14	11/14		11/14
Nagi et al., 2024 [27]	22 teeth, 6–8.5	Reversible pulpitis	Pulpotomy	360, 1800		**360d**				**1800d**				---			
					MTA ProRoot	10/11				8/11							
					TheraCal LC	2/11				2/11							
Zhang et al., 2024 [20]	115 teeth, 11–65	Reversible or irreversible pulpitis	DPC Pulpotomy	90, 180, 360		**90d**		**180d**				**360d**		**90d**	**180d**		**360d**
					iRoot BP Plus	53/54		50/52				47/52		---	---		25/47
					TheraCal LC	54/56		50/54				47/52		---	---		16/47
Iyer et al., 2021 [26]	90 teeth, 15–45	Reversible pulpitis	DPC	30, 90, 180		**30d**		**90d**				**180d**		**180d**			
					Biodentine	27/27		25/27				24/27		20/24			
					MTA Plus	26/26		24/26				24/26		14/24			
					TheraCal LC	27/27		27/27				25/27		18/25			
Peskersoy et al., 2021 [23]	525 teeth 18–42	Reversible pulpitis	DPC	30, 180, 360, 1080		**30d**		**180d**		**360d**		**1080d**		**30d**	**180d**	**360d**	**1080d**
						**C**	**R**	**C**	**R**	**C**	**R**	**C**	**R**				
					Biodentine	97/105	96/105	88/105	90/105	84/105	86/105	83/105	84/105	92/105	90/105	86/105	85/105
					MTA Plus	98/105	104/105	90/105	92/105	90/105	90/105	89/105	90/105	96/105	91/105	89/105	89/105
					TheraCal LC	100/105	101/105	87/105	85/105	77/105	77/105	76/105	77/105	92/105	89/105	76/105	76/105
Velioğlu et al., 2021 [25]	114 teeth 18–45	Reversible pulpitis	DPC	180, 360		**180d**				**360d**				---			
					Biodentine	20/22				20/22							
					MTA Angelus	22/25				21/25							
					TheraCal LC	18/22				18/22							
Gurcan et al., 2019 [24]	295 teeth 4–15	Reversible pulpitis	IPC	180, 360, 540, 720		**720d**											
					MTA ProRoot	50/51								---			
					TheraCal LC	56/63											

**Abbreviations**: **BP**: Bioceramic Putty; **C**: Clinical outcomes; **CEM**: Calcium-Enriched Mixture cement; **D**: Days; **DPC**: Direct Pulp Capping; **IPC**: Indirect Pulp Capping; **MTA**: Mineral Trioxide Aggregate; **LC**: Light-Cured; **R**: Radiographic outcomes; **PT**: Pulpotomy Treatment; **VPT**: Vital Pulp Therapy.

**Table 3:** Summary of findings and certainty of evidence (GRADE) for the comparison between TheraCal LC and non–resin-modified calcium silicate-based materials. Certainty of evidence was assessed using the GRADE approach: ⊕⊕⊕⊕ = high, ⊕⊕⊕○ = moderate, ⊕⊕○○ = low, and ⊕○○○ = very low certainty. Arrows indicate downgrading of the certainty of evidence.

Certainty Assessment							№ of Patients		Effect		Certainty	Importance
№ of Studies	Study Design	Risk of Bias	Inconsistency	Indirectness	Imprecision	Other Considerations	Resin-Modified Calcium Silicate-Based	Control	Relative (95% CI)	Absolute (95% CI)		
**Clinical and radiographic success of vital pulp therapy procedures (IPC, DPC, or pulpotomy) using resin-modified (TheraCal LC) versus non-resin-modified CSMs (follow-up: 90 days; assessed with: Clinical and radiographic success)**												
3	randomised trials	serious	not serious	not serious	serious	none	95/97 (97.9%)	129/135 (95.6%)	**RR 1.01** (0.96 to 1.07)	**10 more per 1000** (from 38 fewer to 67 more)	⨁⨁◯◯ Low	IMPORTANT
**Clinical and radiographic success of vital pulp therapy procedures (IPC, DPC, or pulpotomy) using resin-modified (TheraCal LC) versus non-resin-modified CSMs (follow-up: 180; assessed with: Clinical and radiographic success)**												
4	randomised trials	serious	not serious	not serious	serious	none	105/116 (90.5%)	153/165 (92.7%)	**RR 0.97** (0.90 to 1.04)	**28 fewer per 1000** (from 93 fewer to 37 more)	⨁⨁◯◯ Low	IMPORTANT
**Clinical and radiographic success of vital pulp therapy procedures (IPC, DPC, or pulpotomy) using resin-modified (TheraCal LC) versus non-resin-modified CSMs (follow-up: 360 days; assessed with: Clinical and radiographic success)**												
4	randomised trials	serious	not serious	not serious	serious	none	78/99 (78.8%)	124/138 (89.9%)	**RR 0.89** (0.69 to 1.13)	**99 fewer per 1000** (from 279 fewer to 117 more)	⨁⨁◯◯ Low	IMPORTANT
**Clinical and radiographic success of direct pulp capping using resin-modified (TheraCal LC) versus non-resin-modified CSMs (follow-up: 90 days; assessed with: Clinical and radiographic success)**												
2	randomised trials	serious	not serious	not serious	serious	none	41/41 (100.0%)	76/81 (93.8%)	**RR 1.05** (0.98 to 1.14)	**47 more per 1000** (from 19 fewer to 131 more)	⨁⨁◯◯ Low	IMPORTANT
**Clinical and radiographic success of direct pulp capping using resin-modified (TheraCal LC) versus non-resin-modified CSMs (follow-up: 180 days; assessed with: Clinical and radiographic success)**												
2	randomised trials	serious	not serious	not serious	serious	none	43/49 (87.8%)	90/100 (90.0%)	**RR 0.99** (0.88 to 1.11)	**9 fewer per 1000** (from 108 fewer to 99 more)	⨁⨁◯◯ Low	IMPORTANT
**Clinical and radiographic success of direct pulp capping using resin-modified CSM (TheraCal LC) versus non-resin-modified controls (follow-up: 360; assessed with: Clinical and radiographic success)**												
2	randomised trials	serious	not serious	not serious	serious	none	29/36 (80.6%)	67/75 (89.3%)	**RR 0.90** (0.76 to 1.08)	**89 fewer per 1000** (from 214 fewer to 71 more)	⨁⨁◯◯ Low	IMPORTANT
**Dentine bridge formation following the use of resin-modified CSM versus non-resin-modified controls (direct pulp capping) (follow-up: 180 days; assessed with: Dentine bridge formation)**												
2	randomised trials	serious	not serious	not serious	serious	none	107/130 (82.3%)	215/258 (83.3%)	**RR 0.99** (0.90 to 1.08)	**8 fewer per 1000** (from 83 fewer to 67 more)	⨁⨁◯◯ Low	IMPORTANT
**Dentine bridge formation following the use of resin-modified CSM versus non-resin-modified controls (DPC and pulpotomy) (follow-up: 360 days)**												
3	randomised trials	serious	not serious	not serious	serious	none	103/166 (62.0%)	226/285 (79.3%)	**RR 0.85** (0.76 to 0.95)	**119 fewer per 1000** (from 190 fewer to 40 fewer)	⨁⨁◯◯ Low	IMPORTANT
**Subgroup meta-analyses of clinical and radiographic success of direct pulp capping using resin-CSM (TheraCal LC) versus Biodentine (follow-up: 90 days; assessed with: Clinical and radiographic success)**												
2	randomised trials	serious	not serious	not serious	serious	none	41/41 (100.0%)	39/41 (95.1%)	**RR 1.04** (0.95 to 1.14)	**38 more per 1000** (from 48 fewer to 133 more)	⨁⨁◯◯ Low	IMPORTANT
**Subgroup meta-analyses of clinical and radiographic success of direct pulp capping using resin-CSM (TheraCal LC) versus Biodentine (follow-up: 180 days; assessed with: Clinical and radiographic success)**												
2	randomised trials	serious	not serious	not serious	serious	none	43/49 (87.8%)	44/49 (89.8%)	**RR 0.99** (0.86 to 1.14)	**9 fewer per 1000** (from 126 fewer to 126 more)	⨁⨁◯◯ Low	IMPORTANT
**Subgroup meta-analyses of clinical and radiographic success of direct pulp capping using resin-CSM (TheraCal LC) versus Biodentine (follow-up: 360 days; assessed with: Clinical and radiographic success)**												
2	randomised trials	serious	not serious	not serious	serious	none	29/36 (80.6%)	34/36 (94.4%)	**RR 0.86** (0.71 to 1.03)	**132 fewer per 1000** (from 274 fewer to 28 more)	⨁⨁◯◯ Low	IMPORTANT

## Data Availability

The original contributions presented in the study are included in the article, further inquiries can be directed to the corresponding author.

## Abbreviations

The following abbreviations are used in this manuscript:

Bis-GMA	Bisphenol A-glycidyl methacrylate
CEM	Calcium-enriched mixture cement
CI	Confidence interval
DPC	Direct pulp capping
DSPP	Dentine sialophosphoprotein
Exp_HAA	Experimental hydroxyethyl acrylamide-based material
GRADE	Grading of Recommendations Assessment, Development and Evaluation
CSM	Calcium silicate-based material
HEMA	2-Hydroxyethyl methacrylate
I^2^	I-squared statistic (heterogeneity measure)
IPC	Indirect pulp capping
MTA	Mineral trioxide aggregate
NRM-CSM	Non-resin-modified calcium silicate-based material
PEGDMA	Poly(ethylene glycol) dimethacrylate
PICO	Population, Intervention, Comparator, Outcome
PRISMA	Preferred Reporting Items for Systematic Reviews and Meta-Analyses
RevMan	Review Manager
RM-CSM	Resin-modified calcium silicate-based material
RoB 2	Revised Cochrane Risk of Bias Tool for Randomised Trials
ROS	Reactive oxygen species
RR	Risk ratio
UDMA	Urethane dimethacrylate
VPT	Vital pulp therapy
